# Biejiajian Pill Ameliorates Diabetes-Associated Atherosclerosis through Inhibition of the NLRP3 Inflammasome

**DOI:** 10.1155/2022/9131178

**Published:** 2022-06-02

**Authors:** Yu Fu, Jiayao Yuan, Feng Sang, Mingyi Shao, Shuxun Yan, Leilei Li, Rong Zhu, Zhongrui Wang

**Affiliations:** ^1^Department of Endocrinology, The First Affiliated Hospital of Henan University of Chinese Medicine, Zhengzhou 450000, China; ^2^School of First Clinical, Henan Univerity of Chinese Medicine, Zhengzhou 450000, China; ^3^Department of Key Laboratory of Viral Diseases Prevention and Treatment of Traditional Chinese Medicine of Henan Province, The First Affiliated Hospital of Henan University of Chinese Medicine, Zhengzhou 450000, China; ^4^Department of Gastroenterology, The First Affiliated Hospital of Henan University of Chinese Medicine, Zhengzhou 450000, China

## Abstract

**Objective:**

To research the efficacy of Biejiajian pill (BJJ) on diabetes-associated atherosclerosis and explore its subsequent mechanisms.

**Methods:**

Diabetes-associated atherosclerosis (AS) was established in apolipoprotein E knockout (ApoE^**−/−**^) mice using high-fat diet and streptozotocin. Atorvastatin (ATV, 10 mg/kg/day) or BJJ-L (BJJ low-dose, 0.9 g/kg/day), BJJ-M (BJJ medium-dose, 1.8 g/kg/day), and BJJ-H (BJJ high-dose, 3.6 g/kg/day) were administered to diabetic ApoE^**−/−**^ mice for 12 continuous weeks. The normal control group consisted of 10 male C57BL/6J mice. Atherosclerosis plaques, vascular endothelial function, fasting blood glucose, lipid metabolism, inflammatory factors, NLRP3 inflammasome expression, and mitochondria and autophagy changes were evaluated.

**Results:**

The atherosclerotic lesions areas in the aortas were analyzed through Oil Red O and H&E staining, and they were reduced in the BJJ-H and BJJ-M groups. In the BJJ group, endothelin-1 (ET-1) levels were decreased, whereas endothelial nitric oxide synthase (eNOS) was increased. Fasting blood glucose levels in the BJJ and ATV groups were gradually decreased. Lipid metabolism parameters such as TG, TC, and LDL-C were reduced, while HDL-C was elevated in BJJ groups. The serum IL-1*β* and IL-18 were decreased under BJJ therapy. The aortic mRNA and protein expressions of NF-*κ*B, TXNIP, NLRP3, ASC, caspase-1, and IL-1*β* were inhibited in BJJ-H and BJJ-M groups, especially in the BJJ-H group. Electron microscopy revealed an increase in autophagy in each treatment group.

**Conclusions:**

The findings reveal that BJJ could alleviate diabetic atherosclerosis in diabetic ApoE^**−/−**^ mice by inhibiting NLRP3 inflammasome.

## 1. Introduction

As a result of urbanization and aging, diabetes mellitus (DM) has become a global epidemic. Macrovascular complication is the main clinical outcome and primary cause of disability and mortality among diabetic individuals [1, 2]. The pathological manifestation of the diabetic macrovascular disease is accelerated AS, which mainly involves the aorta, coronary artery, cerebral artery, and peripheral artery. Compared with nondiabetics, atherosclerosis (AS) in diabetic patients has the characteristics of high incidence, young age of onset, rapid disease progression, and multiple organs involved simultaneously [3]. Although the pathogenesis of diabetic atherosclerosis is very complicated and has not yet been elucidated, inflammation-mediated serum lipid accumulation and oxidation, vascular endothelial cell (VEC) damage, macrophages and vascular smooth muscle cell (VSMC) responses are considered to play an important role [4–6].

Evidence is accumulating that nucleotide-binding oligomerization domain-like receptor protein 3 (NLRP3) inflammasomes contribute to diabetic atherosclerosis onset and development [7]. NLRP3, apoptosis-associated speck-like protein (ASC), and caspase-1 comprise NLRP3 inflammasome, which is involved in innate immunity. In diabetic atherosclerosis, the activation and regulation of NLRP3 inflammasome are affected by damage-associated molecular patterns (DAMPs), including hyperglycemia, hyperlipidemia, and urate crystals. The NLRP3 inflammasome induces active interleukin (IL)-1*β* and IL-18, which have been demonstrated to aggravate VEC damage, monocyte adhesion and infiltration, VSMC proliferation, and plaque vulnerability in AS [8, 9]. Furthermore, increasing evidence has demonstrated that inhibiting or silencing the NLRP3 gene can delay the progression of atherosclerotic lesions [10, 11]. Autophagy has been demonstrated to suppress NLRP3 inflammasome, although this effect is disturbed by hyperglycemia. As a result, autophagy has increasingly emerged as a novel focus for modulating NLRP3 inflammasome in diabetic atherosclerosis therapy [12, 13]. Consequently, downregulation of NLRP3 inflammasome may prevent and treat diabetic atherosclerosis.

Biejiajian pill (BJJ) was first recorded in the traditional Chinese medicine classics Synopsis of Golden Chamber, and it has been clinically applied over the past 1800 years to treat cirrhosis and liver cancer in China. In recent years, BJJ has been found to have antiatherosclerotic potential. A randomized controlled trial was used to demonstrate that BJJ can increase the blood flow velocity in the lower limbs, reduce triglyceride (TG), total cholesterol (TC), and low-density lipoprotein cholesterol (LDL-C), and alleviate the symptoms of lower limb pain and numbness in lower extremity arteriosclerosis obliterans of diabetic patients [14]. Furthermore, BJJ protects VEC, limits VSMC proliferation and migration, and stabilizes plaques in diabetic atherosclerosis rats by regulating blood lipids, reducing inflammatory factors, lowering serum endothelin, and increasing serum nitric oxide levels [15–17].

Although BJJ has a clinical effect on diabetic atherosclerosis, the relationship between BJJ and NLRP3 inflammasome remains unknown. This work explored the influences of BJJ in diabetes-associated AS and the potential mechanisms, notably with respect to NLRP3 inflammasome.

## 2. Methods

### 2.1. Animals

The mice utilized in this research were 50 male ApoE^−/−^ mice and 10 male C57BL/6J mice (6 weeks old, bodyweight 18–21 g) from Vital River Laboratory Animal Technology Co., Ltd. (certificate no. SCXK (Beijing) 2016–0006, China). The mice were maintained in a light/dark cycle pathogen-free laboratory (18–26°C ambient temperature; 55% relative humidity). The animal cages, water bottles, beddings, and animal rooms were replaced or sterilized periodically. The mice were allowed to adapt for 7 days. The Institutional Animal Care and Use Committee of the First Affiliated Hospital of Henan University of Chinese Medicine approved the experimental protocol (No. 2018012).

### 2.2. Diabetic Atherosclerosis Mouse Model and Grouping

ApoE^−/−^ mice induced by high-fat diet (HFD) to develop spontaneous atherosclerotic lesions were frequently employed as an atherosclerotic animal model in previous studies [18]. This mice model of atherosclerosis was selected for use in this experiment. After 7 days of accommodation, ApoE^−/−^ mice were given HFD (79.85% standard chow, 15% fat, 5% egg yolk powder, and 0.15% cholesterol), while the C57BL/6J mice were given a standard diet. All animal diets were provided by Zhengzhou Jinshui District Carnival Laboratory Materials Management Department. Subsequently, to induce diabetes mellitus, ApoE^−/−^ mice (13 weeks old, bodyweight 26–30 g) received an intraperitoneal injection of streptozotocin (130 mg/kg; Sigma, MO, USA) after a fast. Citrate buffer (0.1 mol/L) in an equivalent volume was injected intraperitoneally into the C57BL/6J mice. After 1 week, the Accu-Chek glucometer (Roche Diagnostic Products (Shanghai) Co., Ltd.) was applied to measure blood glucose obtained from the tail. Model success was defined as random blood glucose >16.7 mmol/L [18]. Thus, 50 diabetic mice were divided randomly into 5 groups: DM control (DM), BJJ low-dose (BJJ-L), BJJ medium-dose (BJJ-M), BJJ high-dose (BJJ-H), and atorvastatin (ATV) (*n* = 10/group). The normal control group (Con) consisted of ten C57BL/6J mice with random blood glucose levels <7.0 mmol/L.

### 2.3. Drug Administration

After the diabetes-associated atherosclerosis model was developed, doses of 0.9, 1.8, and 3.6 g/kg/day BJJ (3 g/bag, Sinopharm Group Zhong Lian Pharmaceutical Co., Ltd., no. Z42020772) were administered through gavage to the BJJ-L, BJJ-M, and BJJ-H groups. BJJ is comprised of 23 herbal medicines, including Biejiajiao (Trionycis Carapax), Ejiao (Colla Corii Asini), Fengfang (Vespae Nidus), Shufuchong (Armadillidium), Tubiechong (Eupolyphaga Steleophaga), Qianglang (Liinnaeus), Xiaoshi (Saltpeter), Chaihu (Bupleuri Radix), Huangqin (Scutellariae radix), Banxia (Pinelliae Rhizoma), Dangshen (Codonopsis radix), Ganjiang (Zingiberis Rhizoma), Houpo (Magnoliae Officinalis cortex), Guizhi (Cinnamomi Ramulus), Baishao (Paeoniae Radix Alba), Shegan (Belamcandae Rhizome), Taoren (Persicae Semen), Mudanpi (Moutan Cortex), Dahuang (Rhei Radix et Rhizoma), Lingxiaohua (Campsis Flos), Tinglizi (Descurainiae Semen), Shiwei (Pyrrosiae Folium), and Qumai (Dianthi Herba). The dosage was calculated using well-mixed BJJ powders. Atorvastatin (10 mg/kg/day, 20 mg/tablet, Pfizer Pharmaceutical Co., Ltd., no. H20051407) was given to the ATV group. The Con and DM control groups received the same amounts of distilled water by oral gavage (10 ml/kg/day). All drugs were dissolved in distilled water. The mice were treated for 12 weeks.

### 2.4. Blood Collection and Tissue Specimens

Intraperitoneal of 3% sodium pentobarbital (45 mg/kg) was used to anesthetize the mice after 12 weeks of treatment and a 12 h fast. Blood samples were obtained from the orbital venous plexus, left for 2 hours at room temperature before being centrifuged for 10 minutes at 4000 r/min at 4°C, and then stored at −80°C. After orbital blood collection, the chest cavity and abdominal cavity were exposed, and the left ventricle was perfused with cold phosphate-buffered saline (PBS), after which the aorta was isolated under a surgical microscope and rinsed with cold PBS immediately. Each aortic tissue sample was divided into 2 sections. A portion of the upper aorta was preserved in paraformaldehyde (4%) for hematoxylin-eosin (H&E) staining, and another portion was fixed in glutaraldehyde (2.5%) for electron microscopy observation. The lower half of the aorta was immediately placed in liquid nitrogen and used for Western blot and rRT-PCR analysis. In addition, some intact aortic tissues, including the thoracic aorta and abdominal aorta, were isolated for Oil Red O staining.

### 2.5. Metabolic Measurement

Before drug administration, tail vein fasting blood glucose and bodyweight were recorded monthly. The serum high-density lipoprotein cholesterol (HDL-C), TG, TC, and LDL-C levels were determined by a fully automatic animal biochemical analyzer (Mindray Medical, BS-200). IL-1*β*, IL-18, endothelin-1 (ET-1), and endothelial nitric oxide synthase (eNOS) levels in serum were analyzed using an ELISA kit (Elabscience Biotechnology, Wuhan, China) following the manufacturer's instructions.

### 2.6. Atherosclerotic Lesion Area Quantification

For optical microscope observation (Nikon Eclipse, E100), the upper sections of the aortas were removed from 4% paraformaldehyde solution and embedded. 5 *μ*m sections were cut and stained using H&E. After the intact aortas were removed from the mice with the adventitial fat excised under a stereomicroscope (Olympus, SZ61), they were placed in 4% paraformaldehyde solution and fixed for more than 24 h. Afterward, the tissue was washed twice with PBS and unfolded along the longitudinal axis with ophthalmic dissection scissors. The unfolded aortas were then stained for 15 minutes with saturated Oil Red O, differentiated in 75% alcohol three times, and observed with an optical microscope (Nikon Eclipse, E100). A trained observer who was blinded to the experiment utilized Image-Pro Plus 6.0 to quantify the lesion areas (Media Cybernetics Inc.).

### 2.7. Transmission Electron Microscope Analysis

The aortic tissues fixed in 2.5% glutaraldehyde were cut into l mm × l mm × l mm pieces, fixed, postfixed, dehydrated, and infiltrated according to standard electron microscope sample preparation methods and then observed with a transmission electron microscope (Hitachi HT7700).

### 2.8. rRT-PCR Analysis

To extract total mRNA from the aortic tissue, TRIzol (15596018, Invitrogen) was employed, and the manufacturer's instructions were followed. Reverse transcription of RNA was carried out using a kit (4368814, Invitrogen), following the manufacturer's instructions to prepare cDNA. The rRT-PCR was performed using a SYBR Green Mix kit (A25742, Invitrogen), and the primer sequences are given in Table 1. A fluorescence quantitative PCR instrument was used for 40 amplification cycles at 95°C for 5 minutes, 95°C for 15 seconds, and 60°C for 1 minute. The standard curves of the target genes were generated and then normalized to endogenous reference genes. The relative mRNA expression was calculated using the 2−^△△Ct^ method.

### 2.9. Western Blot Analysis

Aorta tissues were homogenized in radioimmunoprecipitation assay (RIPA; Solarbio, R0010, Beijing) lysis buffer. Equal amounts of protein (40 *μ*g) were extracted from the total aorta and separated by 10% SDS–polyacrylamide gel electrophoresis (Solarbio, P1200, Beijing). The proteins were then transferred to polyvinylidene fluoride (PVDF; Millipore, ISEQ00010, USA) membranes and blocked with 5% skim milk powder (BD, 232100). The following primary antibodies were incubated with PVDF membranes at 4°C overnight: NF-*κ*B antibody (1 : 2000, GeneTex, GTX102090), anti-thioredoxin interacting protein (TXNIP) antibody (EPR14774) (1 : 1000, Abcam, ab188865), NLRP3 antibody (1 : 1000, GeneTex, GTX64347), anti-ASC antibody (1 : 200, Abcam, ab175449), caspase-1 antibody (14F468) (1 : 1000, GeneTex, GTX14367), IL-1*β* antibody (1 : 2000, GeneTex, GTX55675), and *β*-actin antibody (1 : 5000, GeneTex, GTX109639). The PVDF membranes were then extensively rinsed and incubated at room temperature with secondary antibodies (1 : 5000, Proteintech, SA00001-1, SA00001-2, Wuhan) for 1 h. A biomolecular imager (Fuji, LAS-4000, MINI, Japan) and QualityOne were used to scan and quantitate the membranes. All experiments were repeated 3 times.

### 2.10. Statistics

SPSS 20.0 was used to calculate significant differences. Mean ± standard deviation (SD) was used to present the data, *t*-tests were used to compare the differences before and after, and one-way ANOVA was employed for group differences. The significance level is taken as *P* < 0.05.

## 3. Results

### 3.1. BJJ Reduced Atherosclerotic Lesion in Diabetic ApoE^−/−^ Mice

**Figure 1:**
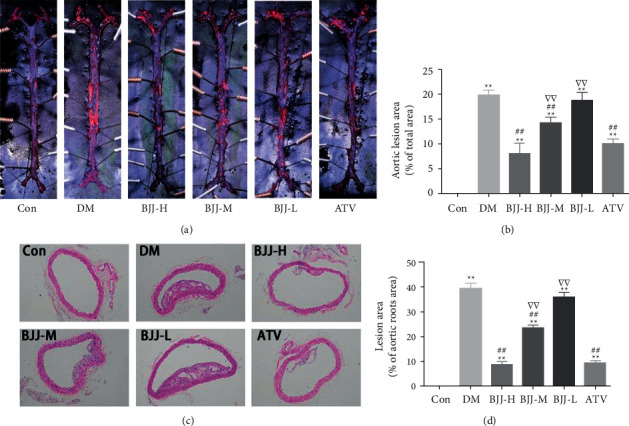
BJJ reduced atherosclerotic lesion in diabetic ApoE^−/−^ mice. (a) Aortic Oil Red O staining. (b) Area percentage of the lesion in the aortas. (c) H&E staining of aortic roots. (d) Area percentage of the lesion in aortic roots. ^*∗∗*^*P* < 0.01 versus the Con group; ^##^*P* < 0.01 versus the DM group; ^▽▽^*P* < 0.01 versus the ATV group. Values presented as mean ± SD (*n* = 3 per group for Oil Red O staining; *n* = 7 per group for H&E staining).

Oil Red O staining of the aorta and H&E staining of aortic roots were performed to investigate whether BJJ could attenuate diabetic ApoE^−/−^ mouse atherosclerotic lesion formation (Figures 1(a) and 1(c)). Compared to the control group, atherosclerotic lesions of the diabetic ApoE^−/−^ groups were larger (*P* < 0.01). Aortic lesions in the groups of BJJ-M, BJJ-H, and ATV were smaller than the DM control (*P* < 0.01). Importantly, the ATV and BJJ-H groups did not have significant differences in aortic lesion areas (*P* > 0.05, Figures 1(b) and 1(d)). Therefore, BJJ treatment of diabetic ApoE^−/−^ mice reduced aortic lesion formation in a dose-dependent manner.

As shown by H&E staining, the Con group's aortic walls were homogenous in thickness, and there were relatively few atherosclerotic lesions. Varying degrees of thickened aortic intimal and wall, newly developed fibrous cap, and evident atherosclerotic lesions were found in the other five groups. The diabetic group had obvious cholesterol crystal production, and the cholesterol crystals were greatly reduced after BJJ treatment (Figure 1(c)).

### 3.2. Changes in Blood Glucose and Bodyweight after BJJ Therapy

**Figure 2:**
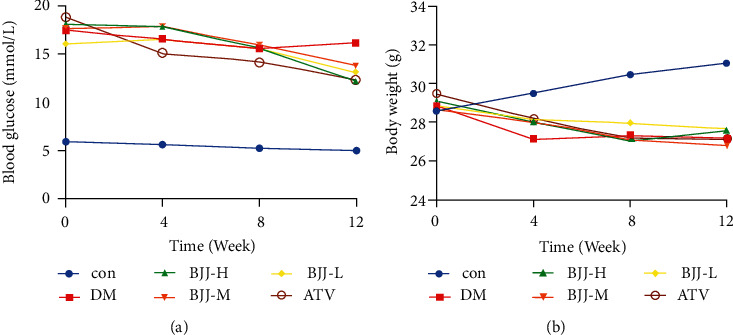
Dynamic body glucose and weight of mice. (a) Blood glucose of mice. (b) Bodyweight of mice. The blood glucose and bodyweight levels were assessed at 0, 4, 8, and 12 weeks of treatment. Values presented as mean (*n* = 10 per group).

Blood glucose and bodyweight were recorded to investigate whether BJJ had an impact on diabetes. The diabetic ApoE^−/−^ mice had higher fasting blood glucose levels than the control group mice at all time periods (*P* < 0.01). After 12 weeks, the ATV and BJJ groups had lower fasting blood glucose levels than the DM control group (*P* < 0.01) (Figure 2(a)). The baseline bodyweights between the groups showed no significant variations (*P* > 0.05). The diabetic ApoE^−/−^ mice gradually lost weight after 4 weeks of treatment. However, BJJ or ATV therapy had no effect on the bodyweight loss of diabetic ApoE^−/−^ mice (*P* > 0.05) (Figure 2(b)).

### 3.3. Changes in Serum Lipid Metabolism after BJJ Therapy

**Figure 3:**
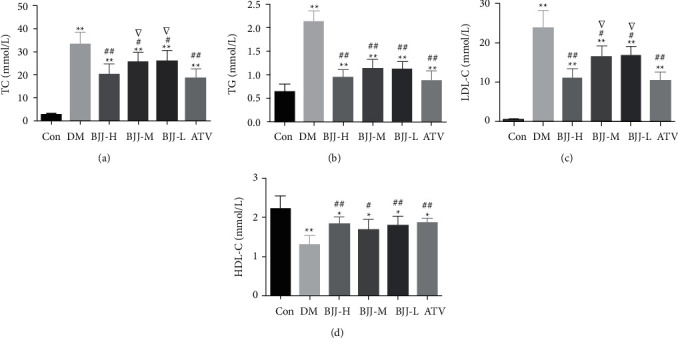
BJJ improved blood serum lipid metabolism in diabetic ApoE^−/−^ mice. (a) TC. (b) TG. (c) LDL-C. (d) HDL-C. ^*∗∗*^*P* < 0.01, ^*∗*^*P* < 0.05 versus the Con group; ^##^*P* < 0.01, ^#^*P* < 0.05 versus the DM group; ^▽^*P* < 0.05 versus the ATV group. Values presented as mean ± SD (*n* = 10 per group).

To investigate whether BJJ influences plasma lipids, the TC, TG, LDL-C, and HDL-C levels were evaluated. The TC, TG, and LDL-C levels in diabetic ApoE^−/−^ mice were increased than the control group (*P* < 0.01). The BJJ and ATV groups had lower TG, TC, and LDL-C levels than the DM control group (*P* < 0.05). HDL-C levels in diabetic ApoE^−/−^ mice were lower than the control group (*P* < 0.05). Both the BJJ and ATV groups had higher HDL-C levels than the DM control group (*P* < 0.05). The BJJ-H group had a similar impact as ATV in regulating serum lipids (*P* > 0.05) (Figure 3).

### 3.4. Changes in Serum Vascular Endothelial Factors and Inflammatory Cytokines after BJJ Treatment

**Figure 4:**
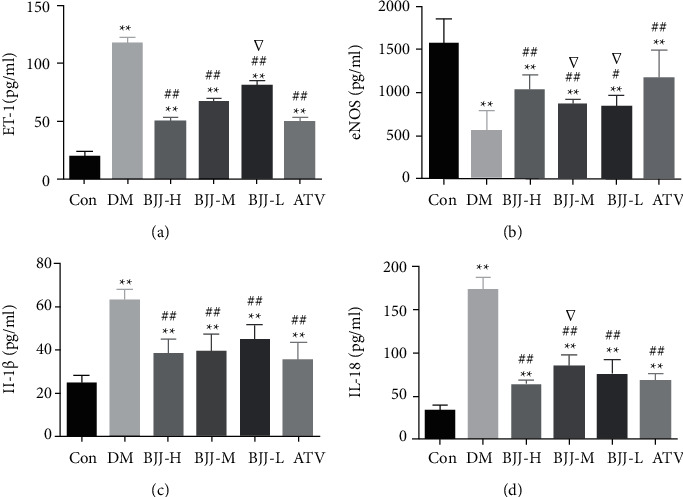
BJJ alleviated vascular endothelial factors and inflammatory cytokines in diabetic ApoE^−/−^ mice. (a) ET-1. (b) eNOS. (c) IL-1*β.* (d) IL-18. ^*∗∗*^*P* < 0.01 versus the Con group; ^##^*P* < 0.01, ^#^*P* < 0.05 versus the DM group; ^▽^*P* < 0.05 versus the ATV group. Values presented as mean ± SD (*n* = 10 per group).

The levels of serum ET-1, eNOS, IL-1*β*, and IL-18 were measured to investigate whether BJJ reduces diabetic atherosclerosis-induced vascular endothelial dysfunction and inflammation. The DM control, BJJ, and ATV groups had higher ET-1 and lower eNOS levels than the control group (*P* < 0.01). All treatment groups had reduced ET-1 levels and elevated eNOS levels than the DM group (*P* < 0.05). The DM control, BJJ, and ATV groups had elevated IL-1*β* and IL-18 levels than the control group (*P* < 0.01). All treatment groups had lower levels of IL-1*β* and IL-18 than the DM group (*P* < 0.01). There were no significant changes in ET-1, eNOS, IL-1*β*, or IL-18 levels between the BJJ-H and ATV groups (*P* > 0.05) (Figure 4).

### 3.5. BJJ Downregulated NLRP3/Caspase-1/IL-1*β* Pathway mRNA Expression in Diabetic ApoE^−/−^ Mouse Aortas

**Figure 5:**
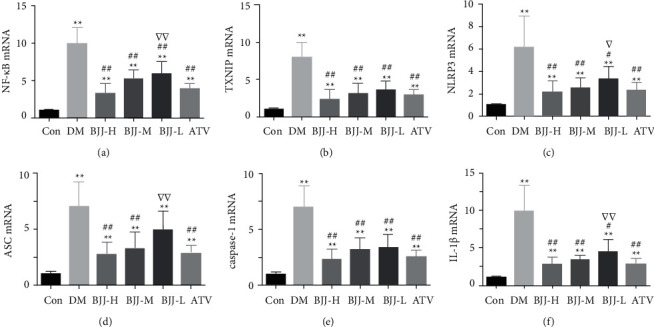
BJJ downregulated NF-*κ*B, TXNIP, NLRP3, ASC, caspase-1, and IL-1*β* mRNA expression in diabetic ApoE^−/−^ mouse aortas. (a) NF-*κ*B mRNA level determined by rRT-PCR. (b) TXNIP mRNA. (c) NLRP3 mRNA. (d) ASC mRNA. (e) Caspase-1 mRNA. (f) IL-1*β* mRNA. ^*∗∗*^*P* < 0.01 versus the Con group; ^##^*P* < 0.01, ^#^*P* < 0.05 versus the DM group; ^▽▽^*P* < 0.01, ^▽^*P* < 0.05 versus the ATV group. Values expressed as mean ± SD (*n* = 7 per group).

The aortic mRNA levels of NF-*κ*B, TXNIP, NLRP3, ASC, caspase-1, and IL-1*β* were measured using rRT-PCR assays to investigate whether BJJ downregulates NLRP3 inflammasome. The DM control, BJJ, and ATV groups had higher mRNA levels of NF-*κ*B, TXNIP, NLRP3, ASC, caspase-1, and IL-1*β* than the control group (*P* < 0.01). The BJJ-M, BJJ-H, and ATV groups had lower mRNA expressions of NF-*κ*B, TXNIP, NLRP3, ASC, caspase-1, and IL-1*β* than the DM control group (*P* < 0.01). BJJ-M and BJJ-H had the same effect as ATV (*P* > 0.05) (Figure 5).

### 3.6. BJJ Downregulated NLRP3/Caspase-1/IL-1*β* Pathway Protein Expression in Diabetic ApoE^−/−^ Mouse Aortas

**Figure 6:**
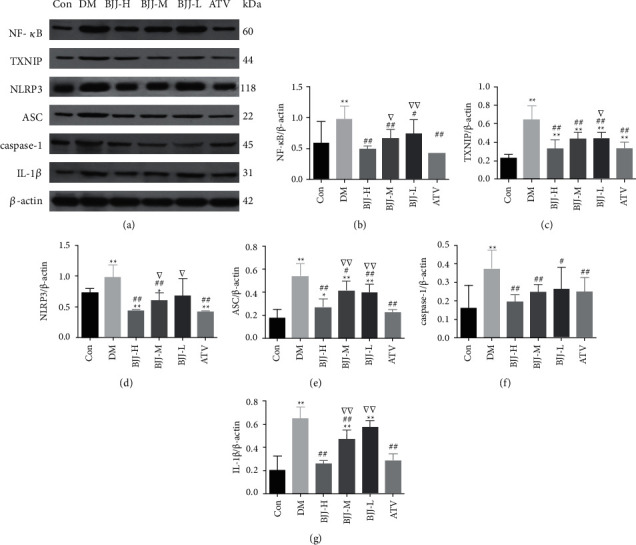
BJJ downregulated NF-*κ*B, TXNIP, NLRP3, ASC, caspase-1, and IL-1*β* protein expression in diabetic ApoE^−/−^ mouse aortas. (a) NF-*κ*B, TXNIP, NLRP3, ASC, caspase-1, and IL-1*β* protein expression measured by Western blot in the Con, DM, BJJ-H, BJJ-M, BJJ-L, and ATV groups. (b) NF-*κ*B protein. (c) TXNIP protein. (d) NLRP3 protein. (e) ASC protein. (f) caspase-1 protein. (g) IL-1*β* protein. ^*∗∗*^*P* < 0.01, ^*∗*^*P* < 0.05 versus the Con group; ^##^*P* < 0.01, ^#^*P* < 0.05 versus the DM group; ^▽▽^*P* < 0.01, ^▽^*P* < 0.05 versus the ATV group. Values expressed as mean ± SD (*n* = 7 per group).

The aortic protein levels of NF-*κ*B, TXNIP, NLRP3, ASC, caspase-1, and IL-1*β* were examined using Western blot to investigate whether BJJ impacts NLRP3 inflammasome. In the DM control group, NF-*κ*B, TXNIP, NLRP3, ASC, caspase-1, and IL-1*β* protein expressions were elevated than the control group (*P* < 0.01). In the BJJ-M, BJJ-H, and ATV groups, the NF-*κ*B, TXNIP, NLRP3, ASC, caspase-1, and IL-1*β* protein expressions were lower than the DM control group (*P* < 0.05). BJJ-H had the same impact as ATV (*P* > 0.05) (Figure 6).

### 3.7. BJJ Stimulated Aortic Endothelial Cell Autophagy in ApoE^−/−^ Mice

**Figure 7:**
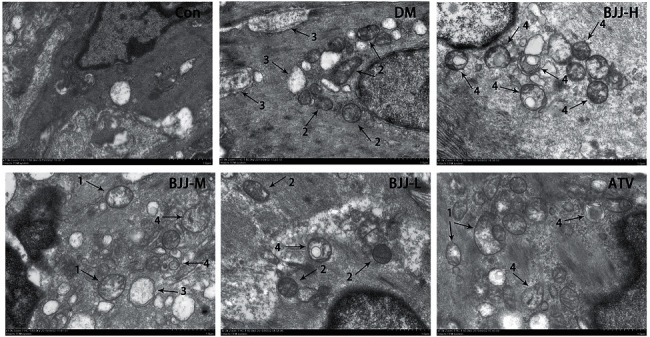
BJJ improved autophagy in diabetic ApoE^−/−^ mice. Representative transmission electron microscopy of autophagy in aortic tissues in the Con, DM, BJJ-H, BJJ-M, BJJ-L, and ATV groups. Transmission electron microscopy images were observed with a Hitachi HT7700 (7000×). Scale bar: 1.0 *μ*m.

To investigate whether BJJ could induce autophagy, the aortas were observed by transmission electron microscopy (Figure 7). In the control group, the mitochondrial membrane structures were intact, and the mitochondrial ridges were clear. In the diabetic ApoE^−/−^ mice of each group, mitochondrial swelling (Figure 7, arrow 1), mitochondrial crista destruction (Figure 7, arrow 2), and increased mitochondrial vacuolization (Figure 7, arrow 3) were observed. Compared with the DM control group, the mitochondrial swelling, mitochondrial cristae destruction, and mitochondrial vacuolization in each treatment group tended to be reduced. Moreover, autophagy was observed in each diabetic ApoE^−/−^ mouse group (Figure 7, arrow 4), especially in each treatment group.

## 4. Discussion

BJJ is a traditional Chinese compound prescription used to treat cirrhosis, fibrosis, and cancer of the liver in the clinic [19–21]. Recently, BJJ was shown to have an antiatherosclerotic effect [22, 23]. Moreover, previous studies by our team confirmed that BJJ has a certain effect on diabetic atherosclerosis rats [15–17], but its exact mechanism remains to be explored. This study revealed that BJJ could inhibit NLRP3 inflammasome and alleviate diabetes-associated atherosclerosis. The results also support the anti-inflammatory effect of BJJ in treating diabetes-associated atherosclerotic vascular disease.

According to a previous study, BJJ improves endothelial function and stabilizes plaques in diabetic atherosclerosis rats [16, 17]. The antiatherosclerotic effect of BJJ in mice with STZ and HFD-induced diabetic atherosclerosis was confirmed in this study. BJJ effectively inhibited and reversed the progression of vascular plaques in ApoE^−/−^ mice, which was supported by the pathological morphology results, such as Oil Red O and H&E staining. Furthermore, BJJ reduced serum ET-1 and increased the level of eNOS in diabetic ApoE^−/−^ mice. These results showed that BJJ could alleviate atherosclerosis and improve vascular endothelial function in diabetic ApoE^−/−^ mice, consistent with previous studies [17].

It is widely recognized that chronic low-level inflammation caused by hyperglycemia and dyslipidemia is a key pathological mechanism that exacerbates diabetic atherosclerosis [24]. After 12 weeks of BJJ therapy, the expressions of FBG, TC, TG, and LDL-C were reduced, while HDL-C was elevated, confirming our earlier research [17]. The IL-1*β* and IL-18 generated by NLRP3 inflammasome have been shown to participate in the progression of diabetic atherosclerosis [18, 25]. In diabetic ApoE^−/−^ mice, serum IL-1*β* and IL-18 levels decreased after 12 weeks, demonstrating that BJJ had an anti-inflammatory effect. These results suggest that BJJ regulates serum FBG and lipids and reduces the release of inflammatory cytokines.

Priming and activation are two signals necessary for NLRP3 inflammasome canonical activation. During the priming signal, the stimulation of hyperglycemia and ox-LDL leads to the phosphorylation of NF-*κ*B. NF-*κ*B is essential to upregulate the NLRP3, pro-caspase-1, and pro-IL-1*β* transcription [26, 27]. During the activation signal, oxidative stress generated by hyperglycemia causes TXNIP and thioredoxin (Trx) to dissociate. Then, TXNIP binds to NLRP3 and stimulates NLRP3 inflammasome formation [28]. In this study, the BJJ treatment group had lower NF-*κ*B and TXNIP mRNA and protein expression. This shows that BJJ may be involved in priming signal to inhibit NLRP3 inflammasome activation. Furthermore, the antioxidative effect of BJJ may be connected to the inhibition of NLRP3 inflammasome. The active NLRP3 inflammasome cleaves IL-1*β* and triggers an inflammatory response after NLRP3, ASC, and caspase-1 protein assembly [25]. Meanwhile, BJJ treatment suppressed mRNA and protein expression of NLRP3, ASC, caspase-1, and IL-1*β* in the aortas of diabetic ApoE^−/−^ mice. These findings suggest that BJJ may inhibit NLRP3 inflammasome by participating in its priming and activation. However, the underlying mechanism needs further exploration.

Autophagy can remove abnormal mitochondria through autophagosomes and negatively regulate NLRP3 inflammasome activation [29]. Hyperglycemia can damage mitochondria directly given a lack of antioxidant capacity. The mtDNA and mtROS released by damaged mitochondria act as DAMPs, further stimulating NLRP3 inflammasome activation [30]. The PI3K inhibitor 3-methyladenine (3 MA) has been shown to inhibit autophagy, resulting in increased mtROS generation and NLRP3/caspase-1/IL-1*β* pathway activation [31]. According to the results of transmission electron microscopy, increased autophagy and decreased mitochondria damage were observed in diabetic ApoE^−/−^ mice, especially in the BJJ treatment group. It is hypothesized that BJJ could regulate autophagy to alleviate NLRP3 inflammasome activation.

The problems with this research and future study directions are as follows: first, BJJ is comprised of 23 kinds of traditional Chinese medicine, and its diverse medicine types make it difficult to determine which active ingredients play a key role in the treatment. Second, it is only speculated that the mechanism by which BJJ negatively regulates NLRP3 inflammasome is related to lowering serum glucose and regulating serum lipids, and additional research is required. Third, due to the lack of ROS detection in the experimental design, it has not been determined whether BJJ can negatively regulate NLRP3 inflammasome activation by reducing ROS in aortic tissue. Fourth, although BJJ can increase autophagy and inhibit NLRP3 inflammasome activation, it has yet to be determined whether there is a crosstalk link. We plan to conduct an in-depth study of BJJ treatments for ApoE^−/−^ mice with diabetic atherosclerosis and use more precise research designs and comprehensive methods to verify our hypothesis. Furthermore, the use of high-performance liquid chromatography and other techniques to separate the chemical components and identify the active ingredients of BJJ is urgently needed. In addition, the potential mechanism will be further verified by exploring the effects of BJJ-treated serum on human umbilical vein endothelial cells or macrophages exposed to hyperglycemia.

## 5. Conclusions

Collectively, this study demonstrated that by suppressing NLRP3 inflammasome, BJJ might reduce atherosclerosis and improve the vascular endothelial cells function in diabetic ApoE^−/−^ mice. This might be connected to the autophagy regulation of BJJ. This study contributes to the application of BJJ to diabetes-associated atherosclerotic patients.

## Figures and Tables

**Table 1:** The primer sequences of the rRT-PCR.

Gene	Primer	Sequences
NF-*κ*B	mNF-*κ*B-F	TGCGATTCCGCTATAAATGCG
	mNF-*κ*B-R	ACAAGTTCATGTGGATGAGGC

TXNIP	mTxnip-F	GGCCGGACGGGTAATAGTG
	mTxnip-R	AGCGCAAGTAGTCCAAAGTCT

NLRP3	mNlrp3-F	ATTACCCGCCCGAGAAAGG
	mNlrp3-R	CATGAGTGTGGCTAGATCCAAG

ASC	mASC-F	GACAGTGCAACTGCGAGAAG
	mASC-R	CGACTCCAGATAGTAGCTGACAA

Caspase-1	mcasp1-F	AATACAACCACTCGTACACGTC
	mcasp1-R	AGCTCCAACCCTCGGAGAAA

IL-1*β*	mIL-1*β*-F	GAAATGCCACCTTTTGACAGTG
	mIL-1*β*-R	TGGATGCTCTCATCAGGACAG
*β*-Actin	mACT*β*-F	GTGACGTTGACATCCGTAAAGA
	mACT*β*-R	GCCGGACTCATCGTACTCC

## Data Availability

The data used to support the findings of this study are available from the corresponding author upon request.
